# Impact of night-shift work on the prevalence of erosive esophagitis in shipyard male workers

**DOI:** 10.1007/s00420-016-1130-x

**Published:** 2016-04-29

**Authors:** Tae Heum Chung, Jiho Lee, Moon Chan Kim

**Affiliations:** Department of Family Medicine, University of Ulsan College of Medicine, Ulsan University Hospital, 877 Bangeojinsunwhando-Ro, Dong-Gu, Ulsan, 44033 Korea; Department of Occupational and Environmental Medicine, University of Ulsan College of Medicine, Ulsan University Hospital, 877 Bangeojinsunwhando-Ro, Dong-Gu, Ulsan, 44033 Korea

**Keywords:** Night-shift work, Prevalence, Erosive esophagitis, Male workers

## Abstract

**Purpose:**

Whether night-shift work is a risk factor for gastroesophageal reflux disease is controversial. The aim of this study was to investigate the association between night-shift work and other factors, and erosive esophagitis.

**Methods:**

A cross-sectional study with 6040 male shipyard workers was performed. Esophagogastroduodenoscopic examination and a survey about night-shift work status, lifestyle, medical history, educational status, and marital status were conducted in all workers. The odds ratios of erosive esophagitis according to night-shift work status were calculated by using the logistic regression model.

**Results:**

The prevalence of erosive esophagitis increased in the night-shift workers [odds ratio, 95 % confidence interval: 1.41 (1.03–1.94)]. According to multiple logistic regression models, night-shift work, obesity, smoking, and alcohol consumption of ≥140 g/week were significant risk factors for erosive esophagitis. By contrast, *Helicobacter pylori* infection was negatively associated with erosive esophagitis.

**Conclusion:**

Night-shift work is suggested to be a risk factor for erosive esophagitis. Avoidance of night-shift work and lifestyle modification should be considered for prevention and management of gastroesophageal reflux disease.

## Introduction

After the industrial revolutions, the concept of shift work emerged, and the type of work was divided into day and night shifts. In a recent population survey, 18 % of the US labor force works in some form of shift work (Mcmenamin 2007). Among the companies in South Korea, 20 % have adopted shift work and 80 % of those companies that have adopted shift work have adopted a 12-h day shift (Son et al. 2008). Working at night can affect health in various manners because it disrupts the circadian rhythm (Knutsson 2003). Almost all physical functions, including the control of appetite and digestion, are regulated by the circadian rhythm (Knutsson 2010).

Gastroesophageal reflux disease (GERD) is a condition developed when the reflux of stomach contents causes troublesome symptoms and/or complications (Vakil et al. 2006). Its prevalence is known to be 10–20 % in the Western world and <5 % in Asia. The most common symptoms are heartburn, regurgitation, and dysphagia. GERD symptoms cause a large burden among employers because of the high absence rate and poor performance (Locke et al. 1997).

Recent studies have indicated that disruption of the circadian system, such as due to shift work, is an important contributory factor in numerous important pathological conditions, including gastrointestinal diseases (Erren and Reiter 2009). Although many studies have investigated the association between shift work and gastrointestinal disorders, these have focused on peptic ulcer disease or unspecific gastrointestinal symptoms (Lundell et al. 1999). A strong evidence links shift work to peptic ulcer disease (Knutsson 2003). However, only one study investigated the association between shift work and GERD; this study used a questionnaire to diagnose GERD (Li et al. 2008). To date, therefore, no study has been published that has examined the possible association between night-shift work and GERD by using endoscopy. Erosive esophagitis (EE) is a complication of GERD that can be easily detected on endoscopy and can be used as an objective criterion for the diagnosis of GERD (Vakil et al. 2006). In this study, we assessed the impact of night-shift work and other factors on the prevalence of EE among shipyard male workers in South Korea, to clarify the association between night-shift work and GERD.

## Methods

### Participants and procedures

Data were obtained from 6040 shipyard workers who were at least 30 years old and underwent a routine health checkup that included esophagogastroduodenoscopy (EGD) at the Ulsan University Hospital Heath Promotion Center (UUH-HPC) in Ulsan, South Korea, between March 2012 and February 2013. In South Korea, the law mandates that all workers in big companies such as those in the shipyard industry must undergo an annual health checkup, including health surveys. During the study period, 6505 subjects were examined, of whom 271 had missing data pertaining to their characteristics and 105 had a history of gastrectomy or cancer, or a current cancer. These 376 subjects were excluded. A total of 26 subjects who worked night shifts for less than 1 year and 63 subjects who worked night shifts on 8-h shifts were also excluded. Of the 6040 shipyard workers eligible, 5577 worked on daytime and 463 worked on rotating night shift by twelve-hour length, working during 8 am to 8 pm for 1 week and then during 8 pm to 8:00 am alternately. The study protocol was approved by the institutional review board of UUH.

### Data collection

Subjects who visited the UUH-HPC to undergo a health checkup were asked to complete a questionnaire about their night-shift work status, lifestyle, medical history, educational status, and marital status. Night-shift status included work duration and night-shift work duration. Lifestyle data included alcohol intake and smoking status. Medical history data included current diseases and medication. Height, weight, and waist circumference (WC) were measured by trained nurses, and a serological test for *Helicobacter pylori* was performed. EGD was performed by experienced endoscopists.

Information about lifetime working years of rotating night shifts was gathered. Body weight and height were measured with the subjects barefoot and wearing light indoor clothing, consistent with the standard protocol. Body mass index (BMI) was defined as body weight in kilograms divided by the square of the height in meters. WC was measured at the midpoint between the lowest ribs and the ileac crest.

Smoking status was scored as “never smoked,” “former smoker,” and “current smoker.” Alcohol consumption was calculated in grams per week. Educational status was scored as ≤9 years (i.e., middle school graduates), 10–12 years (i.e., high school graduates), and ≥13 years (i.e., college graduates). Marital status was classified as either married or single.

*Helicobacter pylori* infection was determined based on the IgG antibody titer for *H. pylori*, determined using the immunochemical luminescence method with DPC Immulite 2000 (DPC, Los Angeles, USA). We defined positivity for *H. pylori* infection if the IgG antibody titer was ≥1.1 U/mL.

EE was defined as the presence of endoscopically detectable mucosal breaks according to the Los Angeles (LA) classification system (Lundell et al. 1999). To maximize the specificity of diagnosis of EE for our analyses, we considered subjects who had endoscopic findings of LA-A, LA-B, LA-C, and LA-D, and excluded those who had minimal change.

### Statistical analyses

We tabulated the possible risk factors for EE and the general information of workers according to the presence of night-shift work. In addition, to determine the difference in mean and distribution of night-shift work according to variables, the Student’s *t* test and Chi-squared test were used, respectively. The association between the influences of the variables and EE was analyzed with logistic regression. The odds ratios (ORs) of EE were calculated by using the multiple logistic regression model, including age, BMI, smoking status, alcohol consumption, educational level, marital status, and *H. pylori* infection. We tested the interaction between night-shift work and variables with respect to their effect on EE. All statistical analyses were performed using the STATA 10.0 software (STATA Corporation, College Station, TX, USA).

## Results

Most of the characteristics of the workers who had worked on night shifts were similar to those of the workers who had never worked night shifts (Table 1). However, the workers with night-shift work were less likely to have received education for ≥13 years (*P* < 0.001).

**Table 1:** Characteristics of the 6040 male shipyard workers according to night-shift work status

Characteristics	Night shift (−)(*n* = 5578)	Night shift (+) (*n* = 462)	*P* value*
Age (year)			
Mean (SD)	50.9 (6.4)	51.0 (6.4)	0.813
Number (%)			0.825
30–44	1299 (23.3)	113 (24.5)	
45–54	2101 (37.7)	169 (36.6)	
55–59	2178 (39.0)	180 (38.9)	
BMI (kg/m^2^)			
Mean (SD)	24.0 (2.6)	23.9 (2.4)	0.198
Number (%)			0.095
Normal (≤22.9)	1904 (34.1)	160 (34.6)	
Overweight (23.0–24.9)	1757 (31.5)	164 (35.5)	
Obesity (≥25.0)	1917 (34.4)	138 (29.9)	
WC (cm)			
Mean (SD)	85.2 (6.7)	84.6 (6.5)	0.086
Number (%)			0.101
Normal (<90)	4370 (78.3)	377 (81.6)	
Obesity (≥90)	1208 (21.7)	85 (18.4)	
Smoking, * n* (%)			0.299
Never	1273 (22.8)	120 (26.0)	
Former	2434 (43.6)	195 (42.2)	
Current	1871 (33.6)	147 (31.8)	
Alcohol consumption (g/week), * n* (%)		0.485	
≤ 69	2788 (50.0)	238 (51.5)	
70–139	1145 (20.5)	84 (18.2)	
≥ 140	1645 (29.5)	140 (30.3)	
Education (y), * n* (%)			<0.001
≤ 9	982 (17.6)	116 (25.1)	
10–12	2385 (42.8)	312 (67.5)	
≥ 13	2211 (39.6)	34 (7.4)	
Marital status, * n* (%)			0.147
Married	5385 (96.5)	440 (95.2)	
Single	193 (3.5)	22 (4.8)	
*Helicobacter pylori*, * n* (%)			0.357
Negative	1934 (34.7)	170 (36.8)	
Positive	3644 (65.3)	292 (63.2)	

*BMI* body mass index, *WC* waist circumference

* The Student’s *t* test was used for continuous variables, and the Chi-square test was used for categorical variables

EE was detected in 8.8 % (530/6040) of the workers who underwent EGD. Table 2 presents the ORs and 95 % confidence interval (CI) of EE with night-shift work adjusted for suspected confounders. In the multiple logistic regression models controlled for multiple covariates, including age, BMI, smoking status, alcohol consumption, educational level, marital status, and *H. pylori* infection, night-shift work was associated with EE (OR 1.41; 95 % CI 1.03–1.94). Obesity, smoking, and alcohol consumption of ≥140 g/week were significantly associated with EE (OR 1.76; 95 % CI 1.40–2.21; OR 1.97; 95 % CI 1.49–2.62; OR 1.51; 95 % CI 1.22–1.87, respectively). In comparison, *H. pylori* infection was negatively associated with EE (OR 0.29; 95 % CI 0.24–0.35). No significant interaction was observed between night-shift work and any of the potential confounding variables.

**Table 2:** Odds ratios (ORs)* of erosive esophagitis (EE) according to night-shift work and associated risk factors among 6040 male shipyard workers

Characteristics	EE (+) (*n* = 530)	EE (−) (*n* = 5510)	Adjusted OR (95 % CI)^*†^
Night-shift work			
No	477 (90.0)	5101 (92.6)	1
Yes	53 (10.0)	409 (7.4)	1.41 (1.03–1.94)
Age (year)			
30–44	139 (26.2)	1273 (23.1)	1
45–54	177 (33.4)	2093 (38.0)	0.89 (0.70–1.14)
55–59	214 (40.4)	2144 (38.9)	1.10 (0.85–1.43)
BMI (kg/m^2^)			
≤ 22.9	135 (25.5)	1929 (35.0)	1
23.0–24.9	164 (30.9)	1757 (31.9)	1.31 (1.03–1.67)
≥ 25.0	231 (43.6)	1824 (33.1)	1.76 (1.40–2.21)
Smoking, * n* (%)			
Never	74 (14.0)	1319 (23.9)	1
Former	221 (41.7)	2408 (43.7)	1.48 (1.12–1.95)
Current	235 (44.3)	1783 (32.4)	1.97 (1.49–2.62)
Alcohol consumption (g/week), * n* (%)			
≤ 69	216 (40.8)	2810 (51.0)	1
70–139	106 (20.0)	1123 (20.4)	1.10 (0.86–1.41)
≥140	208 (39.2)	1577 (28.6)	1.51 (1.22–1.87)
Education (y), * n* (%)			
≤ 9	115 (21.7)	983 (17.8)	1
10–12	219 (41.3)	2478 (45.0)	0.75 (0.58–0.97)
≥13	196 (37.0)	2049 (37.2)	0.82 (0.61–1.08)
Marital status, * n* (%)			
Married	503 (94.9)	5322 (96.6)	1
Single	27 (5.1)	188 (3.4)	1.40 (0.91–2.16)
*Helicobacter pylori*, * n* (%)			
Negative	329 (62.1)	1775 (32.2)	1
Positive	201 (37.9)	3735 (67.8)	0.29 (0.24–0.35)

*BMI* body mass index, *WC* waist circumference

* Using logistic regression

^†^Adjusted for age, body mass index, waist circumference, smoking, alcohol consumption, education level, marital status, and *Helicobacter pylori*

## Discussion

Night-shift work was associated with a 1.41-fold greater increase in risk of EE than day-shift work (OR 1.41; 95 % CI 1.03–1.94) in the male Korean shipyard workers. Obesity, smoking, and alcohol consumption were also positively associated with EE. In comparison, *H. pylori* infection was negatively associated with EE.

In this study, the prevalence of EE was 8.8 %, which is similar to the prevalence rates of 6.7, 7.9, and 7.6 % in previous Korean studies (Kim et al. 2011a, b; Shim et al. 2009; Lee et al. 2011) and 6.4 % in a Chinese study (Zou et al. 2011), but lower than the prevalence rates of 15.5 and 11.8 % in Western populations (Ronkainen et al. 2005; Zagari et al. 2008).

Li et al. conducted a cross-sectional investigation among outpatients in ten hospitals in China in order to elucidate the epidemiology of gastrointestinal reflux disease symptoms (Li et al. 2008). The study used the Chinese version of the reflux disease questionnaire to diagnose GERD. The researchers showed that night-shift work was significantly associated with GERD. The results were compatible with those of the present study. However, as the study was not designed to evaluate the relationship between night-shift work and GERD, night-shift variables were not described. Furthermore, the study population was not representative of a healthy working population.

No plausible pathophysiological mechanism has been proposed to explain the relationship between night-shift work and GERD. A disrupted circadian rhythm may result from desynchronization of the environment, such as shift work, gene polymorphism, or physiological aging (Konturek et al. 2011). The circadian system controls gut motility, gastric acid secretion, maintenance and restoration of the protective mucosal barrier, production of digestive enzymes, and the immunological system of the gastrointestinal tract (Konturek et al. 2011). Klupinska et al. reported that circulating melatonin levels were significantly lower in patients with the erosive form of GERD than in patients with the non-erosive reflux form (Klupinska et al. 2006). Konturek et al. suggested the possible dose-dependent protective role of melatonin in preventing reflux-induced changes in the esophageal mucosa. Melatonin is an important regulator of circadian system (Konturek et al. 2011). Several mechanisms of action of melatonin on GERD were proposed. Melatonin increases reduced esophageal sphincter pressure, blood flow, and anti-inflammatory molecules in the esophageal mucous and decreases gastric acid secretion (Kandil et al. 2010; Konturek and Konturek 2007).

In addition to the disruption of the circadian system, irregular dietary habit, which is common among shift workers, could lead GERD. Yamamichi et al. reported a correlation between GERD and dietary habits such as having dinner a few hours before going to bed, eating midnight snacks, frequently skipping breakfast, and quick eating (Yamamichi et al. 2012). If these dietary habits are the main causes of GERD in night-shift workers, we could prevent and manage GERD by dietary habit modification. Unfortunately, we did not investigate dietary habits in this study.

BMI was not associated with night-shift work in this study. Although some previous studies showed that BMI was positively associated with night-shift work, most of these studies included female subjects such as nurses (Ramin et al. 2015; Peplonska et al. 2015; Amani and Gill 2013), whereas the present study included only male workers. Some studies with male subjects showed no or negative association between BMI and night-shift work (Fesharaki et al. 2014; van Amelsvoort et al. 2004; Nakamura et al. 1997). Further studies to investigate whether interaction between night-shift work and sex exists with respect to their effect on obesity are required. In comparison, BMI was significantly associated with EE in our study as in most previous studies (Hampel et al. 2005; El-Serag et al. 2007). A recent meta-analysis demonstrated a positive association between BMI and the presence of EE, especially in men. Several hypotheses have been proposed to explain the relationship between obesity and EE. Increased intra-abdominal pressure and subsequent esophageal acid exposure, which are hormonal factors related to adiposity, and increased transient lower esophageal sphincter (LES) relaxation were associated with obesity in previous studies (El-Serag et al. 2006, 2007; Wu et al. 2007).

Smoking was not associated with night-shift work in this study. In a recent study with 4685 Koreans, Cho et al. reported that smoking was not associated with night-shift work (Cho et al. 2013). However, most of the previous studies reported a positive association between smoking and night-shift work (Ramin et al. 2015; Fesharaki et al. 2014; Van Amelsvoort et al. 2004; Peplonska et al. 2015; Nicholson et al. 1999). To clarify the cause of the discrepant results, additional studies are needed. However, smoking was significantly associated with EE in the present study. Some previous studies reported a positive relationship between smoking and EE (Chiba et al. 2012; Kim et al. 2011a, b). Cigarette smoking has been reported to prolong esophageal acid clearance times with hyposalivation, provoke acid reflux, and reduce LES pressure (Kahrilas and Gupta 1989; Kahrilas et al. 1990).

In accordance with previous reports, alcohol consumption was significantly associated with EE in our study (Chiba et al. 2012; Kim et al. 2011a, b). Excessive alcohol intake has been reported to promote acid regurgitation by reducing LES pressure and slowing both esophageal motility and gastric emptying (Kaufman and Kaye 1978; Keshavarzian et al. 1990; Mincis et al. 1995).

In comparison, *H. pylori* infection was negatively associated with EE, which is consistent with previous reports (Chung et al. 2011; Kim et al. 2008; Unal et al. 2006; Nordenstedt et al. 2007). These results suggest that *H. pylori*-associated inflammation plays a role in the prevention of the development of EE. However, the association between *H. pylori* and EE is complex and remains incompletely defined.

To the best of our knowledge, this is the first study to evaluate the relationship between night-shift work and EE. The reflux diagnostic questionnaire has some limitation to detect GERD because of its low specificity of 50 % (Li et al. 2008). Hence, instead of a using questionnaire, the present study used endoscopic findings to diagnose GERD.

Our study has several limitations. First, we did not investigate the history of reflux-inducing habits such as supine position after meals and meals before bedtime. In addition, we did not investigate the possibility that dietary habits, including fat, chocolate, and caffeine intakes are associated with GERD. These dietary factors can confound the association between night-shift and EE. Second, inter-operator variability of EGD in the assessment of esophagitis could occur. To minimize this, we used the LA classification, which is the most thoroughly evaluated classification for esophagitis and widely used. Third, the study participants were healthy male workers with the same occupational backgrounds. Therefore, it was difficult to generalize our findings to encompass women or other working groups owing to selection bias. Finally, our study is cross-sectional; hence, we could not investigate the causality between night-shift work and GERD.

Our study revealed that night-shift work is a risk factor for GERD, and avoidance of night-shift work should be considered a primary prevention strategy for GERD. Physicians should pay attention to night-shift work as a risk factor when managing GERD. New studies that take into consideration history of reflux-inducing habits as variables are required to confirm the association between night-shift work and GERD.
